# Enhanced early rehabilitation and pain management with all‐arthroscopic medial patellofemoral ligament reconstruction: A comparative study

**DOI:** 10.1002/jeo2.70291

**Published:** 2025-05-27

**Authors:** Yi‐Fan Song, Hai‐Jun Wang, Xin Yan, Zi‐Jie Xu, Xin‐Jie Wang, Fei Wang, Jia‐Kuo Yu

**Affiliations:** ^1^ Sports Medicine Department, Beijing Key Laboratory of Sports Injuries Peking University Third Hospital Beijing China; ^2^ Institute of Sports Medicine Peking University Beijing China; ^3^ Department of Orthopaedic and Sports Medicine Center, Tsinghua Changgung Hospital Medical Center Tsinghua University Beijing China; ^4^ Department of Joint Surgery The Third Hospital of Hebei Medical University Shijiazhuang China

**Keywords:** arthroscopy, dislocation, knee, patellar

## Abstract

**Purpose:**

The purpose of this study was to evaluate the accuracy of femoral tunnel location, post‐operative pain management, functional rehabilitation and clinical outcomes in medial patellofemoral ligament (MPFL) reconstruction using all‐arthroscopic technique.

**Methods:**

Between 2020 and 2021, 160 patients with recurrent patellar dislocation undergoing MPFL reconstruction were categorized into control (traditional surgery) and study (all‐arthroscopic technique) groups. Femoral tunnel accuracy was assessed via computed tomography scans, pain management, functional rehabilitation, knee range of motion and daily activities were evaluated up to 6 months post‐operatively. Knee function was assessed using Kujala and Lysholm scores at post‐operative 12 months.

**Results:**

Seventy‐one patients in the control group and 69 patients in the study group reached the final follow‐up with no demographic differences. Follow‐up duration was 12.65 ± 0.68 vs 12.77 ± 0.73 months in the control and study groups (*p* = 0.3145). The intra‐class correlation coefficient was excellent (*r* = 0.97). In femoral tunnels, 93.5% in the control group and 92.4% in the study group were correctly localized. In patellar tunnels, 96.1% in the control group and 96.2% in the study group were correctly localized (*p* > 0.9999). Post‐operative strong opioid analgesics were used 25.9 ± 31.0 versus 12.0 ± 22.2 mg/day in the control and study groups (*p* = 0.0016). The pain score was 3.4 ± 1.1 versus 2.7 ± 1.2 in the control and study groups (*p* = 0.0006) during post‐operative functional rehabilitation. Time to resume daily living was 8.2 ± 0.6 versus 7.6 ± 0.6 weeks in the control and study groups (*p* < 0.0001). Time to resume low‐intensity exercise was 12.3 ± 0.6 versus 11.7 ± 0.6 weeks in the control and study groups (*p* < 0.0001). In the more than 1‐year follow‐up, no significant difference was found in the Kujala and Lysholm scores.

**Conclusions:**

The all‐arthroscopic technique for MPFL reconstruction in recurrent patellar dislocation ensures precise femoral tunnel placement. It offers advantages in early post‐operative pain management and functional recovery, enabling faster rehabilitation compared to traditional non‐all‐arthroscopic techniques.

**Level of Evidence:**

Level III.

**Abbreviations**
AT: adductor tubercle
CI: confidence interval
CT: computed tomography
FP: femoral portal
ICC: intraclass correlation coefficient
MPFL: medial patellofemoral ligament
MSP: medial superior portal
NRS: Numeric Rating Scale
PE: posterior edge
PP: patellar portal
ROM: range of motion
SPSS: Statistical Package for the Social Sciences

## INTRODUCTION

Patellar dislocations are a prevalent occurrence, particularly among individuals who engage in regular physical activity, with an annual incidence rate of 23.2 per 100,000 individuals [23]. A comprehensive systematic review encompassing 2558 patients and 35 articles revealed that a substantial majority (94.7%) of individuals with acute lateral patellar dislocations experienced disruption of the medial patellofemoral ligament (MPFL) [16].

MPFL reconstruction has proven to be an effective procedure for addressing recurrent patellar instability, with outcomes generally being better in aligned knees [24, 28]. Moreover, for lower‐extremity malalignment, MPFL reconstruction was often combined with other realignment procedures, such as tibial tubercle osteotomy or trochleoplasty. However, a certain number of complications still existed. The incidence of complications following primary MPFL reconstruction has been reported to range from 0% to 32.3% of cases, including post‐operative objective instability, recurrent dislocation, and arthrofibrosis [10, 14].

Arthrofibrosis is a pathological process marked by scar tissue formation in a joint and surrounding tissues, causing restricted movement and pain, often leading to loss of flexion and/or extension, especially after injury or trauma [4, 9, 15].

Most MPFL reconstruction procedures are performed using non‐arthroscopic techniques, there is a common need for a parapatellar incision to facilitate tunnel localization [1, 6, 21]. This aspect of the procedure may pose a potential risk factor for post‐operative stiffness following MPFL reconstruction. The all‐arthroscopic technique could offer potential advantages over open approach techniques. These include reduced disruption of soft tissues, resulting in less scarring and adhesions, as well as improved cosmetic outcomes [18].

In the previous study, Schottle's point was shown at approximately 8 mm distal to the apex of the adductor tubercle (AT) and 8mm from the posterior edge (PE) [29]. And using anatomic landmarks to locate Schöttle's point without fluoroscopy has been shown to achieve a significantly higher accuracy rate during MPFL reconstruction, with an ideal localization rate of 93.6% [29]. Building upon this established femoral tunnel locating method, an all‐arthroscopic technique has been implemented during MPFL reconstruction with the aim of reducing post‐operative pain and stiffness, as well as facilitating improved functional rehabilitation.

The aim of this study was to evaluate the accuracy of femoral tunnel location and clinical outcomes in MPFL reconstruction using an all‐arthroscopic technique. The hypothesis of this study was that the all‐arthroscopic technique would enable precise femoral tunnel placement and yield advantages in terms of post‐operative pain management and functional rehabilitation, as compared to the traditional non‐all‐arthroscopic technique commonly employed in MPFL reconstruction.

## MATERIALS AND METHODS

This prospective study was conducted in the Peking University Third Hospital Sports Medicine Department. And this study was approved by the Peking University Third Hospital Medical Science Research Ethics Committee (No. 2014099). From January 2020 to December 2021, the consecutive patients at our institution undergoing MPFL reconstruction for recurrent patellar dislocation were enroled. Inclusion criteria: at least two episodes of patellar dislocation, post‐operative 12 months for minimum follow‐up. Exclusion criteria: previous knee surgeries, open physes, trochlear dysplasia (type B to D, according to the classification of Dejour et al.), the tibial tuberosity–trochlear groove distance of greater than 20 mm or patella alta (Insall‐Salvati index >1.2) [5]. And 160 patients were divided into two groups based on a combination of patient preferences and surgeon recommendations. The final decision was documented in the operation notes and used for subsequent analysis. The control group underwent traditional surgical techniques, while the study group underwent the all‐arthroscopic technique. To enhance data availability, patients lost to follow‐up were included in the analysis for femoral tunnel accuracy.

The clinical characteristics of patients were collected from medical records. And post‐operative clinical outcomes, including pain management and functional rehabilitation, were collected through an online platform. Pain medication usage and Numeric Rating Scale (NRS) pain grades were utilized to assess post‐operative pain levels. Knee range of motion (ROM) and activities of daily living were employed to evaluate the progress of rehabilitation. Knee ROM and activities of daily living were used to evaluate rehabilitation process. Pain management, functional rehabilitation, knee ROM, and activities of daily living were assessed up to 6 months after the operation. The patients were instructed how to conduct self‐assessments during their hospitalization and reported their own assessments online every week until post‐operative 6 months. Furthermore, knee function was evaluated using the Kujala and Lysholm scores at post‐operative 12 months.

### Surgical technique

A 2–3 cm incision was performed to expose pes anserinus insertion. Generally, gracilis tendon autograft was harvested with a tendon stripper for reconstruction. The semitendinosus tendon would be taken if the gracilis tendon was too thin or short.

**Figure 1:**
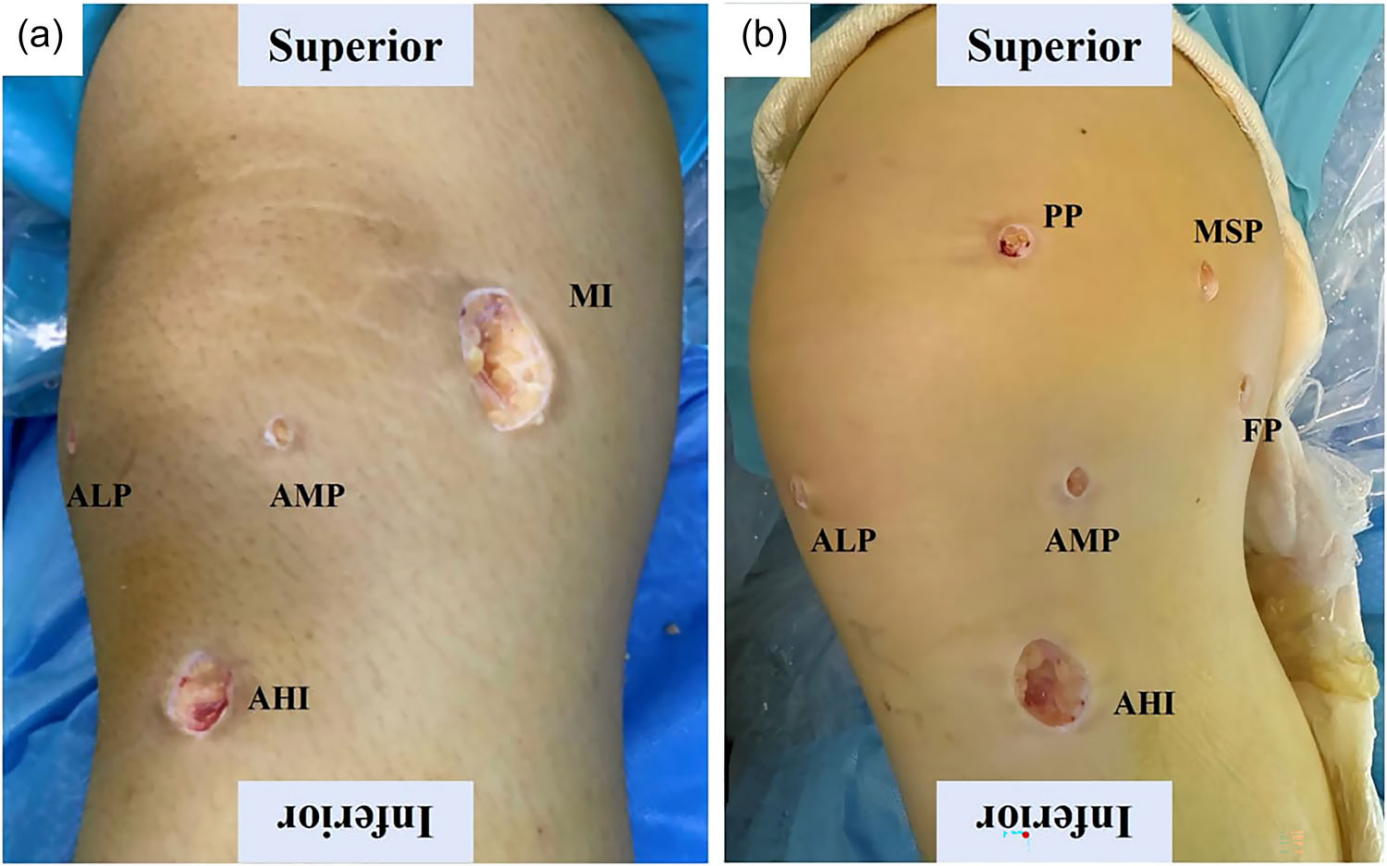
Incisions and arthroscopic portals. (a) Incisions and arthroscopic portals in control group. (b) Incision and arthroscopic portals in study group. AHI, autograft harvesting incision; ALP, anterolateral portal; AMP, anteromedial portal; FP, femoral portal; MI, medial incision; MSP, medial superior portal; PP, patellar portal.

**Figure 2:**
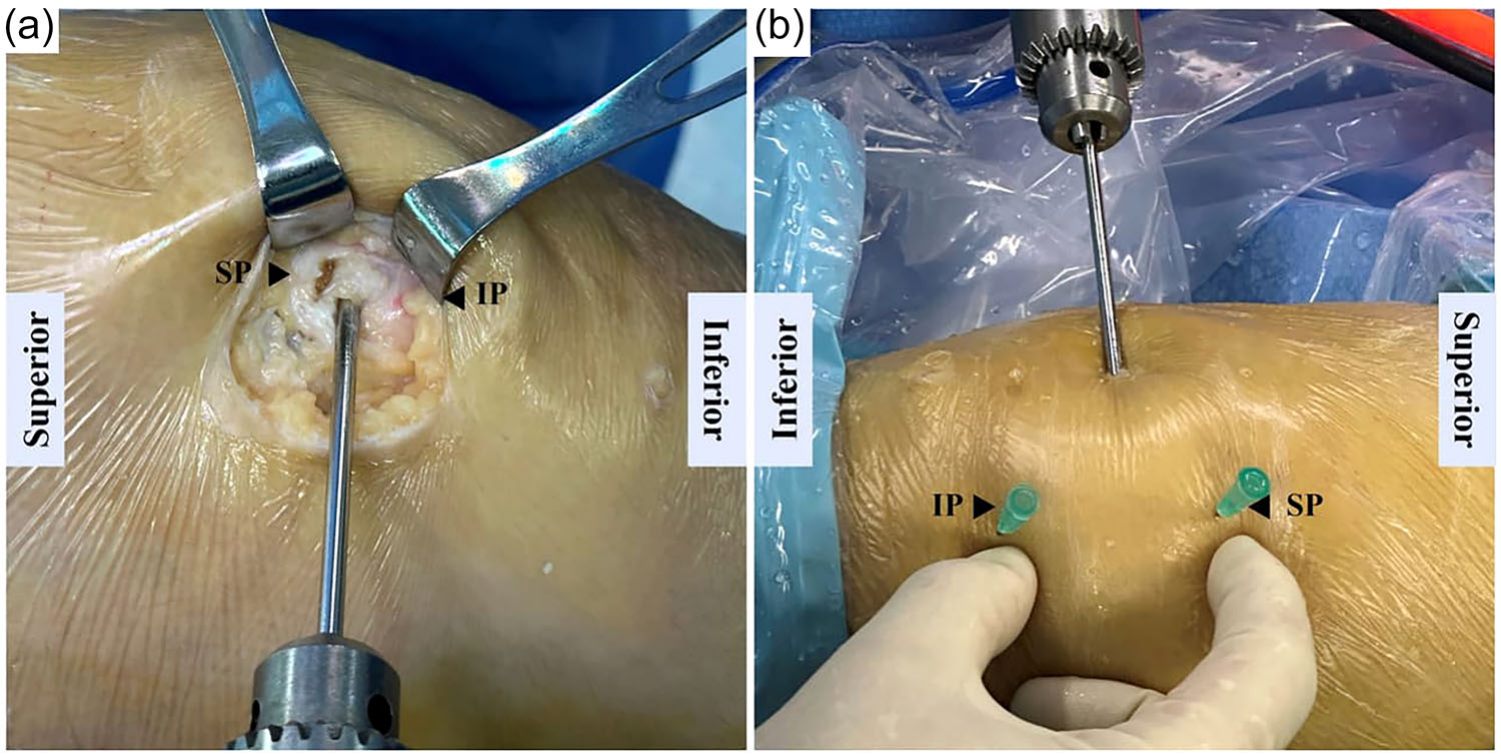
Patellar tunnel locating method. (a) Locating patellar tunnel in control group. (b) Locating patellar tunnel in study group. IP, inferior pole; SP, superior pole.

**Figure 3:**
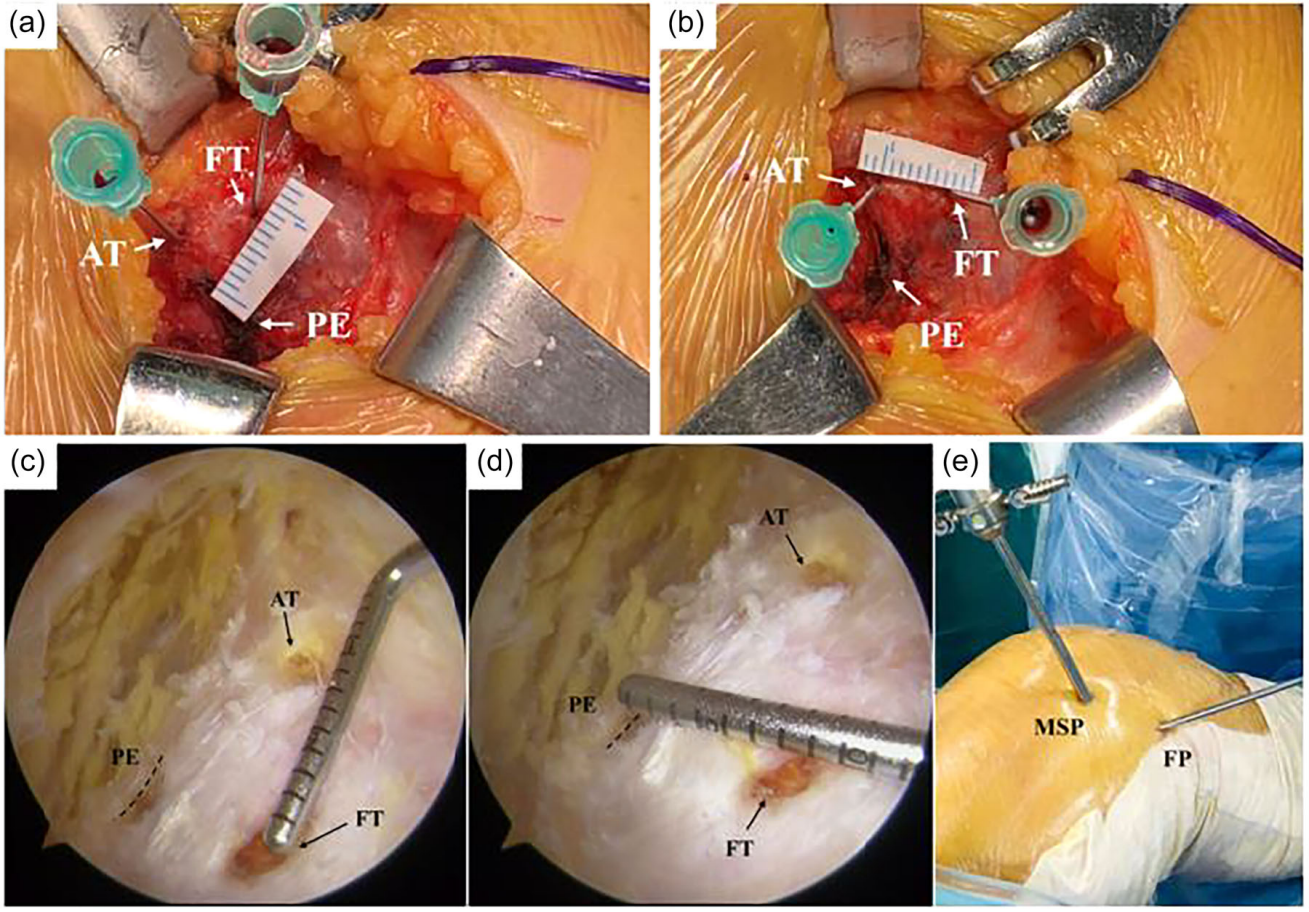
Femoral tunnel locating method. (a and b) Femoral tunnel was located at 8 mm distal to the apex of the AT and 8 mm anterior to the PE in control group. (c and d) Femoral tunnel was located at 8 mm distal to the apex of the AT and 8 mm anterior to the PE in study group. (e) Medial superior portal (MSP) and femoral portal (FP) were used to locate femoral tunnel. AT, adductor tubercle; FT, femoral tunnel; PE, posterior edge.

In the control group, a 3–4 cm incision was made between medial facet of patella and medial condyle. In the study group, femoral portal (FP), patellar portal (PP) and medial superior portal (MSP) were used to locate bone tunnels (Figure 1). The FP was located at 10 mm distal to AT. The PP was located at about 10 mm medial to the medial patella rim. MSP was used as a tunnel locating assistant. A single patellar tunnel (3.5 mm in general) was drilled between the midpoint of the medial border and the superomedial corner of the patella (Figure 2). The femoral tunnel was located at 8 mm distal to the apex of AT and 8 mm anterior to the PE (Figure 3). A proper femoral tunnel was drilled according to the autograft diameter (6 mm in general).

The autograft was placed inside the patellar tunnel, and then the two free ends were pulled into the femoral tunnel between the second and third layers of the medial side of the knee. The knee was cycled several times from full flexion to full extension with the graft under proper tension. And during this procedure, the graft length basically remained isometric. The graft was fixed in the femoral tunnel using absorbable interference screws at 30° knee flexion angle.

### Rehabilitation

The post‐operative rehabilitation objectives are outlined as follows: attaining a knee ROM of 90° at 4 weeks after surgery, progressing to a knee joint ROM of 120° by post‐operative 6 weeks, achieving full knee joint flexion at post‐operative 8 weeks, meeting the functional demands of daily activities by post‐operative 8 weeks, and being able to engage in light physical activities such as jogging or cycling at post‐operative 12 weeks. Both groups followed the same rehabilitation guidelines post‐operatively. A knee brace was used post‐operatively for a period of 6 weeks. The weight‐bearing protocol was as follows: partial weight‐bearing was allowed at 4 weeks post‐surgery, and full weight‐bearing was permitted at 8 weeks, depending on the patient's recovery and progress.

### Computed tomography (CT) and three‐dimensional (3D) measurements

**Figure 4:**
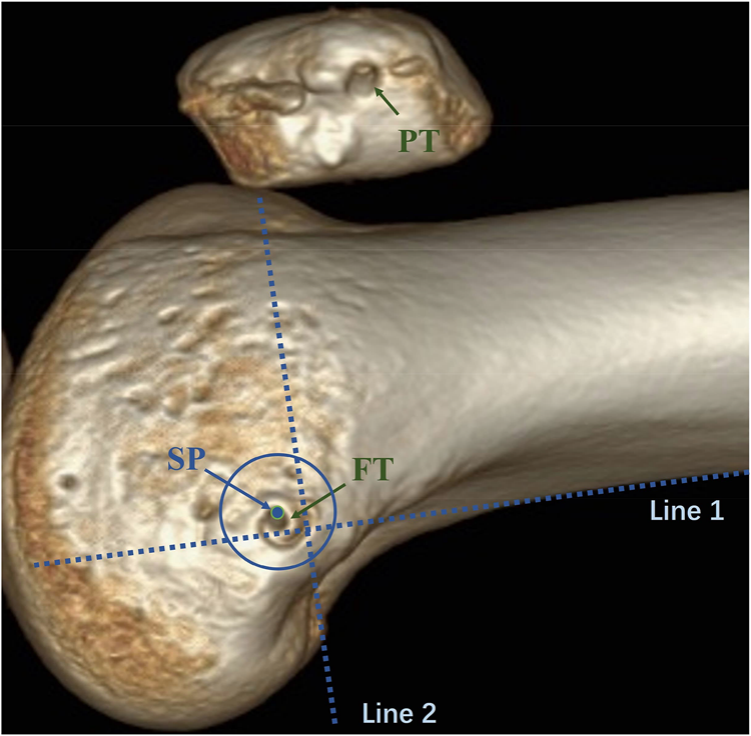
Post‐operative femoral tunnel assessment. The blue dot is Schöttle's point. Line 1 is the extension of the posterior cortical line. Line 2 is the perpendicular line of Line 1 intersecting the posterior origin of the medial femoral condyle. Schöttle's point was located at 1.3 mm anterior to the Line 1, 2.5 mm distal to the Line 2. The red dot is Schöttle's point. The area in the blue circle was the proper zone of the femoral tunnel (8.5 mm in radius). The area in the blue circle was Schöttle's zone (5 mm in diameter). FT, femoral tunnel; PT, patellar tunnel; SP, Schöttle's point.

All patients underwent knee scans using a 64‐row multislice CT scanner (Siemens Healthineers) at 0° knee flexion. Axial plane images with 1.0‐mm slices were acquired and saved as Digital Imaging and Communications in Medicine data. Image reconstruction was performed using an AW VolumeShare 4 workstation monitor (GE HealthCare). A 3D high‐resolution bone surface rendering knee image was obtained in all cases. The 3D images were set so that the posterior portion of the medial femoral condyle and the lateral femoral condyle would fully coincide. These images were projected onto a 2D view, and true lateral views were created [29]. Femoral tunnel was considered to be accurately placed if it completely fell within a virtual circle around Schöttle's point with a radius of 8.5 mm, and considered malpositioned if any part of the tunnel fell outside (Figure 4) [29]. The patellar attachment of the MPFL extends distally from the superomedial corner of the patella over approximately a half of the length of the medial aspect of the patella [2]. The patellar tunnel would be considered to be accurately placed if it was located between superior one third and the middle of patella. Otherwise, the settlement of the tunnel would be regarded as malposition. All the measurements were made by the same radiologist to avoid interobserver error. Each measurement was made two times within a 1‐week interval, and the mean value was acquired to analyze.

### Statistics analysis

In this study, we conducted a follow‐up on two groups of patients, with an initial sample size of 80 per group. During the follow‐up process, we documented the attrition, with 9 patients lost to follow‐up in the control group and 11 in the study group. To assess the potential impact of attrition on the study outcomes, we performed a sensitivity analysis. We compared the complete case analysis, which included all patients (even those with missing data), with the results after multiple imputation. The results indicated that the conclusions drawn from both analytical approaches were consistent, suggesting that patients lost to follow‐up did not significantly affect the current study outcomes.

Mean values were calculated with 95% confidence intervals (CIs). Statistical analysis was performed using the Statistical Package for the Social Sciences (SPSS) 17.0 software package (SPSS Inc.). To evaluate the agreement of measurements, intraclass correlation coefficients (ICCs) were calculated using two‐way mixed‐effects models. ICCs <0.5, 0.5–0.75, 0.76–0.9 and >0.9 were interpreted as poor, moderate, good and excellent reliability, respectively. Demographic data were compared between the two groups using independent sample *t* tests or the chi‐square test. For data that follow a normal distribution, independent samples *t* test was used, while for data that do not follow a normal distribution, Mann–Whitney *U* test was used. A *p* value of <0.05 was considered statistically significant.

## RESULTS

From January 2020 to December 2021, 160 consecutive patients met the inclusion criteria. And 80 patients were enroled in the control and study groups, respectively. Three patients in the control group and one in the study group declined to participate. Six patients of the control group and 10 patients of the study group were lost to follow‐up. Follow‐up duration was 12.65 ± 0.68 versus 12.77 ± 0.73 months in the control and study groups (*p* = 0.3145). The demographic characteristics were shown in Table 1. No significant difference was found between the two groups in age, sex, the side of knee, failure rate, intraoperative and post‐operative complication rate (*p* > 0.05). However, due to the learning curve associated with the all‐arthroscopic surgical technique, the operative time in the study group (60.2 ± 10.8 min) was longer than that in the control group (56.5 ± 4.2 min) (*p* < 0.01). The intra‐class correlation coefficients were excellent for all measures (*r* = 0.97). In the control group, 72 out of 77 femoral tunnels (93.5%) and 74 out of 77 patellar tunnels (96.1%) were considered to be localized in the proper zone (*p* > 0.9999). And in the study group, 73 out of 79 femoral tunnels (92.4%) and 76 out of 79 patellar tunnels (96.2%) were considered to be localized in the proper zone (*p* > 0.9999).

**Table 1:** Demographic characteristics.

	Control	Study	*p*
No. of patients	71	69	
Age (years)	21.8 ± 7.0	23.0 ± 7.5	0.302
Male (%)	23 (32.4)	26 (37.7)	0.596
Left knee (%)	37 (52.1)	34 (49.3)	0.866
BMI	23.8 ± 3.6	23.8 ± 4.0	0.992
Surgical time (min)	56.5 ± 4.2	60.2 ± 10.8	0.009
Failure rate	1.41% (1/71)	1.45% (1/69)	1.000
Intraoperative complication rate (joint loose body)	19.7% (14/71)	18.8% (13/69)	0.877
Post‐operative complication rate	0% (0/71)	0% (0/69)	1.000

Abbreviation: BMI, body mass index.

**Figure 5:**
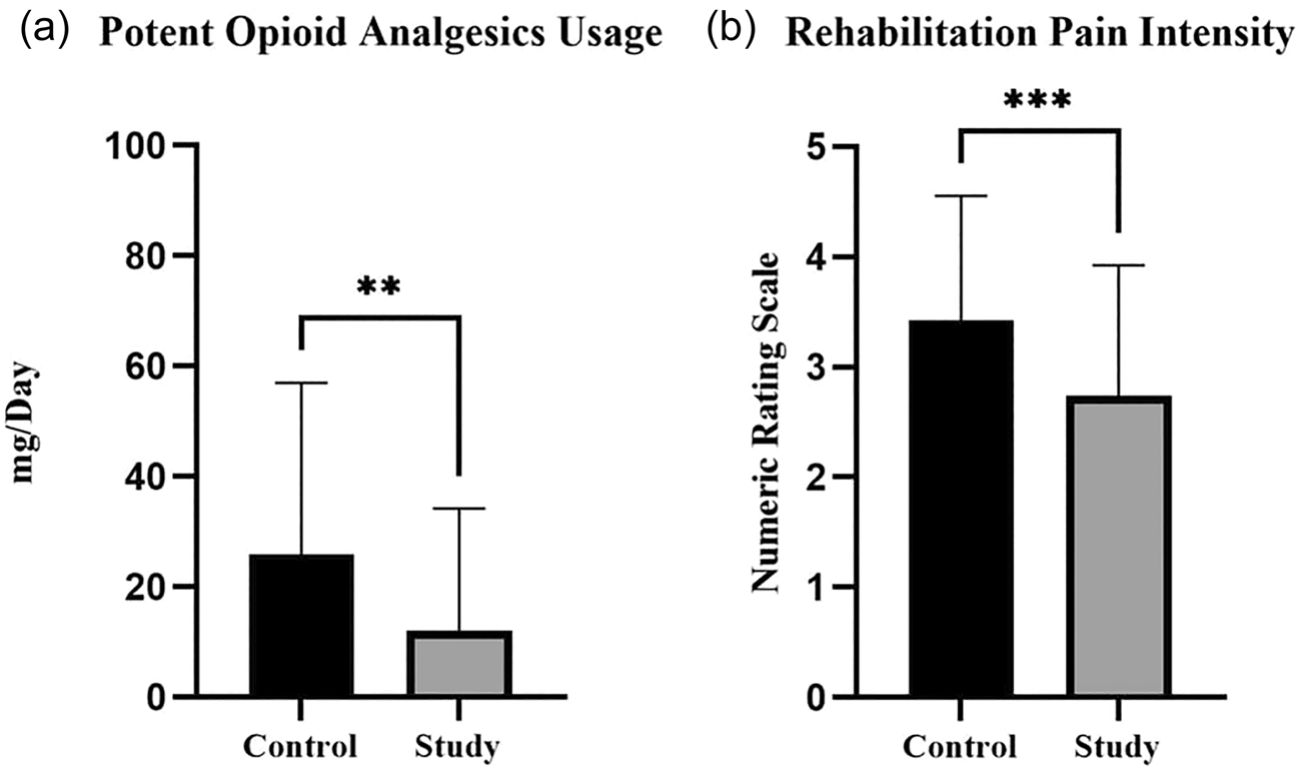
Pain management. (a) Potent opioid analgesics usage. (b) Post‐operative functional rehabilitation (**p < 0.01; ***p < 0.001).

Study group demonstrated advantages in pain management (Figure 5). During the hospitalization, post‐operative potent opioid analgesics usage was significantly reduced in the study group. And during the post‐operative functional rehabilitation, NRS pain score was significantly less in study group (Table 2).

**Table 2:** Pain management.

	Control	Study	*p* (95% confidence intervals)
Potent opioid analgesics (mg/day)	25.9 ± 31.0	12.0 ± 22.2	0.0016 (not applicable)
NRS pain score	3.4 ± 1.1	2.7 ± 1.2	0.0006 (−1.070 to −0.2968)

Abbreviation: NRS, Numeric Rating Scale.

**Figure 6:**
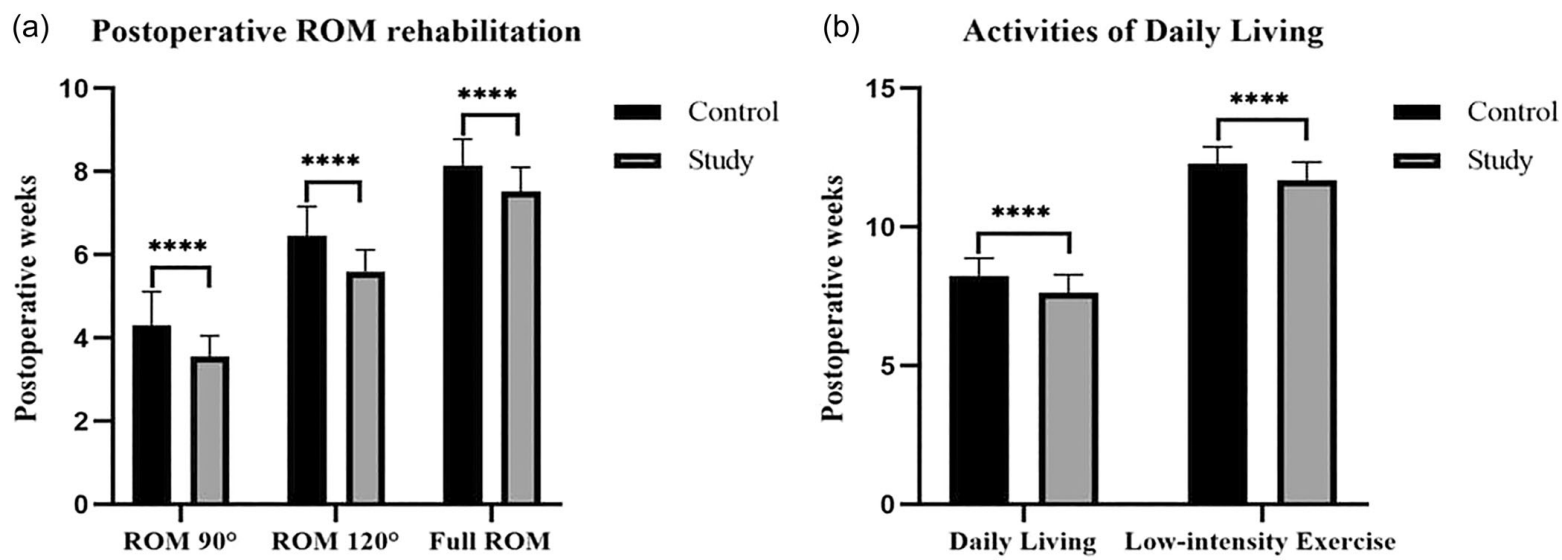
Knee functional rehabilitation progress. (a) Post‐operative ROM rehabilitation, including 90°, 120° and full knee ROM. (b) Post‐operative activities of daily living, including low‐intensity exercise (****p < 0.0001). ROM, range of motion.

In the hand of post‐operative functional rehabilitation, the study group's time‐to‐rehabilitation targets in the study group were significantly reduced in 90°, 120° and full knee ROM. And the post‐operative period of knee function recovery to meet daily life or mild exercise intensity was less in the study group (Table 3, Figure 6). In the more than 1‐year follow‐up, no significant difference was found in Kujala and Lysholm scores (Table 4).

**Table 3:** Knee functional rehabilitation progress.

	Control	Study	*p* (95% confidence intervals)
ROM 90° (weeks)	4.3 ± 0.8	3.6 ± 0.5	<0.0001 (−0.9836 to −0.5346)
ROM 120° (weeks)	6.5 ± 0.7	5.6 ± 0.5	<0.0001 (−1.076 to −0.6648)
Full ROM (weeks)	8.1 ± 0.6	7.5 ± 0.6	<0.0001 (−0.8239 to −0.4143)
Daily living (weeks)	8.2 ± 0.6	7.6 ± 0.6	<0.0001 (−0.8163 to −0.3872)
Low‐intensity exercise (weeks)	12.3 ± 0.6	11.7 ± 0.6	<0.0001 (−0.7970 to −0.3751)

Abbreviation: ROM, range of motion.

**Table 4:** Knee function evaluation.

	Control	Study	*p*
Preoperative Kujala score	60.5 ± 6.5	61.8 ± 6.7	0.235
Post‐operative Kujala score	81.5 ± 12.8	84.3 ± 10.3	0.160
Preoperative Lysholm score	59.7 ± 6.5	59.0 ± 6.4	0.494
Post‐operative Lysholm score	80.3 ± 15.4	84.3 ± 11.5	0.082

## DISCUSSION

The most important finding of the present study was that the all‐arthroscopic technique in MPFL reconstruction for the treatment of recurrent patellar dislocation accurately locates the femoral tunnel and offers advantages in terms of post‐operative early‐stage pain management and functional rehabilitation compared to the traditional non‐all‐arthroscopic technique. The femoral tunnel locating method was based on a previous study, and proper femoral tunnel placement was achieved in 93.6% of patients, locating Schöttle's point without fluoroscopy, which was 8 mm distal to the apex of the AT and 8 mm anterior to the PE [29]. In the present study, the all‐arthroscopic technique similarly yielded proper femoral tunnel location in 73 out of 79 patients, accounting for a rate of 92.4%. Furthermore, the all‐arthroscopic technique exhibited advantages in terms of minimizing incisions and reducing scar tissue formation around the knee joint, thereby resulting in reduced post‐operative pain and improved functional rehabilitation following MPFL reconstruction.

Currently, there are several established techniques for determining the femoral attachment, such as palpation and fluoroscopy methods [7, 17, 19, 20, 25, 26]. However, a prospective study conducted by Servien et al. revealed that only 69% of the 29 femoral tunnels located in an ideal zone were accurately identified using the palpation method [26]. Similarly, a comparison between the palpation method and Schöttle's method conducted by Koenen et al. indicated that only 52% of the femoral tunnels identified by palpation were correctly positioned within the desired zone [17]. In two separate clinical studies, the use of an intraoperative palpation method resulted in the identification of ideal femoral tunnels in 71.3% of 143 knees and 56.2% of 73 knees [11, 12]. One anatomical factor contributing to these limitations is the difficulty in clearly identifying the apex of the flat medial epicondyle. Furthermore, in a previous study conducted by Sanchis et al., it was found that only 38 out of 100 knees had ideal femoral tunnels when using fluoroscopy as the locating method [22]. One possible reason for this discrepancy could be the varying interpretations of the true lateral radiograph, which is crucial for precise fluoroscopic positioning. Some studies have argued that obtaining a true lateral image intraoperatively is often challenging, and measurements taken in the operating room are largely subjective [3, 30]. In a previous study, we discovered that 93.6% of forty‐seven femoral tunnels were considered ideal using an established locating method [29]. Comparing this with the previous palpation methods that relied solely on the AT or the medial epicondyle, which were not easily palpated, the established method using two clearly identifiable landmarks (apex of the AT and PE) demonstrated improved accuracy in positioning [29]. Therefore, these landmarks could be readily identified via arthroscopy.

To date, there has been limited research conducted on the utilization of an all‐arthroscopic technique in MPFL reconstruction [13, 27]. Siebold et al. presented a description of an arthroscopic extraarticular approach to access the MPFL origin on the femur, with subsequent confirmation through lateral fluoroscopy [27]. However, it is important to note that this technique is arthroscopically demanding and more time‐consuming compared to the mini‐open technique, as it adds approximately 15 min to the overall procedure [27]. On the other hand, Hu et al. and Gao et al. described an arthroscopic femoral tunnel placement technique that involves utilizing the midpoint between the AT and medial epicondyle [8, 13]. Neither of these studies reports on the accuracy of femoral tunnel placement, clinical outcomes, or advantages of the techniques. Additionally, the technique described by Siebold et al. does not address the limitation of not being able to obtain an accurate lateral fluoroscopic view intraoperatively [27]. Furthermore, the method proposed by Hu et al. does not guarantee precise localization of the medial epicondyle under arthroscopy [13]. The present study provides evidence that the established femoral tunnel locating method maintains a high level of accuracy when utilized under arthroscopy, comparable to that of the open approach. Furthermore, the study reveals the benefits of this method in terms of post‐operative pain management and functional rehabilitation.

### Limitations

There were several limitations in this study. Firstly, this study is the non‐randomized allocation of patients to the two surgical groups, which was based on a combination of patient preferences and surgeon recommendations. While this approach reflects real‐world clinical decision‐making, it may introduce selection bias, as patient and surgeon preferences could be influenced by factors such as disease severity, comorbidities, or prior treatment history. To address this, we conducted a comprehensive baseline comparison of demographic and clinical characteristics between the groups. Nevertheless, future studies employing randomized designs would be valuable to further validate our results and minimize potential bias. Secondly, it is important to acknowledge the presence of a learning curve inherent to any surgical technique. To address this, we recommend a stepwise approach, starting with the open approach and mastering the femoral tunnel locating method, before progressing to arthroscopic procedures.

## CONCLUSIONS

The all‐arthroscopic technique for MPFL reconstruction in recurrent patellar dislocation ensures precise femoral tunnel placement. It offers advantages in early post‐operative pain management and functional recovery, enabling faster rehabilitation compared to traditional non‐all‐arthroscopic techniques.

## AUTHOR CONTRIBUTIONS

Substantial contributions to the conception or design of the work: Yi‐Fan Song, Hai‐Jun Wang, Xin Yan, Zi‐Jie Xu, Fei Wang and Jia‐Kuo Yu. Acquisition, analysis or interpretation of data for the work: Yi‐Fan Song, Hai‐Jun Wang, Xin Yan and Zi‐Jie Xu. Drafting the work or revising it critically for important intellectual content: Yi‐Fan Song, Hai‐Jun Wang and Xin Yan. Final approval of the version to be published: Yi‐Fan Song, Hai‐Jun Wang, Xin Yan, Zi‐Jie Xu, Xin‐Jie Wang, Fei Wang and Jia‐Kuo Yu. Agreement to be accountable for all aspects of the work in ensuring that questions related to the accuracy or integrity of any part of the work are appropriately investigated and resolved: Yi‐Fan Song, Hai‐Jun Wang, Xin Yan, Zi‐Jie Xu, Xin‐Jie Wang, Fei Wang and Jia‐Kuo Yu.

## CONFLICT OF INTEREST STATEMENT

The authors declare no conflicts of interest.

## ETHICS STATEMENT

All research methodologies involving human participants strictly adhered to the ethical guidelines of the relevant institutional and national review boards. Additionally, these procedures were conducted in line with the principles of the 1964 Helsinki Declaration. This study was approved by the Peking University Third Hospital Medical Science Research Ethics Committee (project number 2014099).

## Data Availability

Data are not available due to ethical restrictions. Due to the nature of this research, participants of this study did not agree for their data to be shared publicly, so supporting data are not available.
